# Diurnal Variation of Islet Autoantibody Titers in Established Type 1 Diabetes Suggests Restricted-Time Sampling Improves Aab Measurement and Detection

**DOI:** 10.21203/rs.3.rs-9328145/v1

**Published:** 2026-05-06

**Authors:** Craig Beam, Linda DiMeglio, Cate Speake, Kathrine Boest-Bjerg, Olivia Pearce, Bashayr Arif Almutairi, Carmella Evans-Molina, F. Susan Wong, Anna E. Long, Kathleen M. Gillespie, Pearson James

**Affiliations:** Homer Stryker MD School of Medicine, Western Michigan University; Indiana University School of Medicine; Benaroya Research Institute; University of Birmingham College of Medicine and Health; University of Bristol Medical School; Cardiff University School of Medicine; Indiana University School of Medicine; Cardiff University School of Medicine; University of Bristol Medical School; University of Bristol Medical School; University of Birmingham College of Medicine and Health

**Keywords:** Type 1 Diabetes, Autoantibodies, Diurnal Variation, Circadian Variation, COSINOR, Radiobinding Assay (RBA), Machine Learning

## Abstract

Diurnal Variation of Islet Autoantibody Titers in Established Type 1 Diabetes Suggests Restricted-Time Sampling Improves Aab Measurement and Detection

Diurnal islet autoantibody (Aab) variation in type 1 diabetes (T1D) remains poorly understood and could hinder efforts to develop, test and refine new therapies. We sought to describe the extent and pattern of diurnal variation in islet autoantibodies and, having found significant variation, translate to the clinical setting.

We conducted two studies in human subjects with established T1D: (1) a prospective study (n = 10) of the range of islet autoantibody and immunoglobulin daily variation within humans and (2) an independent retrospective, cross-sectional study (n = 705) of the effect of time of sample collection on Aab titer and detection in clinical settings.

We found that some individuals have wide Aab variations during the day and which can exceed expected levels of inter-assay variation. For some Aab, this variation followed a circadian pattern. We also found that, in clinical settings, time-restricted sampling can lead to increased IA-2A and ZnT8A detection within specific age-groups.

We conclude that time-restriction can potentially improve the use of Radiobinding-measured Aabs as biomarkers for the development and monitoring of disease-modifying therapies and in developing islet transplantation strategies in established T1D. Investigation of diurnal Aab variation and time restriction is needed in early-stage disease.

## Introduction

Type 1 diabetes (T1D) results from an autoimmune attack on insulin-producing pancreatic beta-cells reducing endogenous insulin production. It is a significant global health burden, with an estimated 8.4 million people worldwide living with the disease in 2021[1]. The mainstay of currently available therapies is limited to ongoing insulin supplementation; however, a new disease-modifying intervention is now clinically available (teplizumab to delay clinical onset for Stage 2 diabetes-i.e., persons having multiple positive autoantibodies (Aab) and impaired glucose tolerance)-and more disease-modifying therapies and improved islet-cell transplantation/replacement strategies are on the horizon. These new therapies are aimed at preserving beta cell function to improve clinical outcomes as twenty percent of Diabetes Control and Complications Trial participants who were within 5 years of clinical diagnosis had significant remaining insulin production (0.2–0.5 pmol/ml). This preserved production was associated with increased normal glycemia and reduced complications.

Pancreatic islet autoantibodies (Aabs) are established biomarkers for type 1 diabetes[3]. The presence of 2 or more autoantibodies is associated with development of T1D at a rate of approximately 70% over 10 years; lifetime risk approaches 100%. Accurate Aab measurement is essential to the development and testing of disease-modifying treatment modalities. Islet Aab can also be useful markers in individuals living with type 1 diabetes, as the Aab presence and number of the beta cell antigens they recognize can be associated with either beneficial or detrimental impacts on endogenous insulin secretion[4–6]. Some have also suggested that Aabs are important biomarkers of therapeutic efficacy, since they can be measured early after treatment; this is particularly relevant to antigen-specific therapies but may be important in other immunotherapies as well [7,8]. A new phase III clinical trial, DIAGNODE-3[9], aims to determine whether intra-lymphatic administration of rhGAD65 preserves insulin secretion and improves glycemia in HLA DR3-DQ2 positive individuals with recently diagnosed type 1 diabetes, and is using the level of GAD65 Aab titer as a biomarker of antigen-specific immune responses to therapy.

Stem cell transplantation is emerging as a promising avenue for treating type 1 diabetes, aiming to restore insulin production by replacing destroyed pancreatic β-cells. Clinical trials have demonstrated the potential of stem cell-derived islet cells in restoring insulin production. For example, the VX-880 therapy (Vertex) showed that persons with type 1 diabetes could achieve glucose-responsive insulin production, leading to reduced or eliminated need for external insulin; albeit with the need for chronic immunosuppressive therapy[10,11]. Some studies have suggested that Aab measurement could be used to both select patients suitable for transplantation and for monitoring resultant auto-and alloimmunity after surgery[12,13]. Thus, maximizing the sensitivity of Aab measurements and minimizing sources of Aab variation may have important implications in the context of transplantation.

In sum, accurate measurement of islet Aabs is necessary to develop, test and evaluate new disease modifying therapies, and to improve the success and safety of stem cell transplantation in persons with established Stage 4 type 1 diabetes (S4T1D). Since Aab precision is affected by variation, it is essential therefore to understand the nature and extent of autoantibody variation and how it can be accounted for when interpreting Aab results. Assay precision has been greatly improved through international Aab assay standardization programs[14,15]; however, although successful in reducing the unwanted assay variation coming from technical variation of the assay itself (“intra-assay” variation) and from differences between laboratories (“inter-laboratory” variation), these programs have not addressed another possible source of variation: variation introduced by blood sampling at different times of day, i.e., “diurnal variation”.

It is established that the human immune system exhibits diurnal variation[16–18]. Our previous work demonstrated that immune populations that are specifically relevant to type 1 diabetes also exhibit diurnal variation[19](includingB-cells, which secrete (auto)antibodies), and we therefore hypothesized that islet autoantibodies might vary diurnally as well.

In this study we sought to determine whether immunoglobulins and islet autoantibodies also exhibit diurnal variation and whether this variation impacts the sensitivity of autoantibody detection in type 1 diabetes. By doing so we hoped to identify times of autoantibody sampling that could increase autoantibody detection and increase the likelihood of success in developing and testing new therapeutic approaches in established type 1 diabetes.

## Results

Diurnal Variation Study

### Immunoglobulin diurnal variation

**Figure 1:**
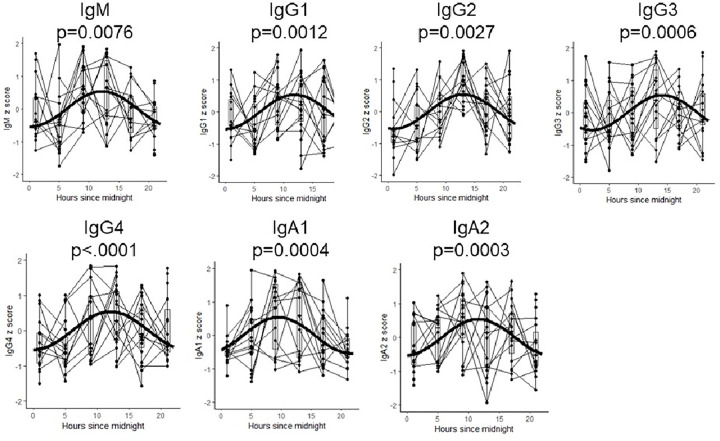
Circadian Immunoglobulin Variation Panels **A-G:** Immunoglobulin titer data from 10 individuals with type 1 diabetes were standardized by “Z-scores” to reduce inter-individual variations and plotted across time and connected by fine lines. Bold superimposed sinusoidal single-phase curves were fit using the COSINOR method and depict circadian patterns with p-values.

Ten individuals (6 females and 4 males, median age 28 years, range 18–40 years) living with type 1 diabetes, (median disease duration 11 years, range: 1–19 years) were enrolled and studied at the Indiana University Clinical Research Center[19]. To determine the extent to which isotype-specific antibodies fluctuated with time of day, we measured immunoglobulin isotypes (IgM, IgG1–4 and IgA1–2) from repeated samples collected every 4-hours over a 24-hour period. All immunoglobulins exhibited statistically significant circadian variation (Fig. 1). Statistically-estimated times of peak immunoglobulin titers are reported in Appendix Table 1. Apart from IgA1, the immunoglobulins tended to reach peak levels around noon (12:00). IgA1 peak titer is estimated to occur approximately at 9:48 (95% confidence interval: 8:00,11:12).

In nine historical controls (4 females, median age 32, range 22–38) only IgM, IgG2 and IgA1 were found to have statistically significant circadian variation. IgM and IgG2 immunoglobulins peaked at times similar to that found in type 1 diabetes (i.e., around noon); however, the peak time for IgA1 in the historical controls was 12:06 (9:24,14:48) which is approximately 3 hours later than the time of peak found in type 1 diabetes participants (9:48). Yet, based on overlapping confidence intervals, this difference in peak times failed to reach statistical significance (Appendix Table 1).

### Islet autoantibody diurnal variation

**Figure 2:**
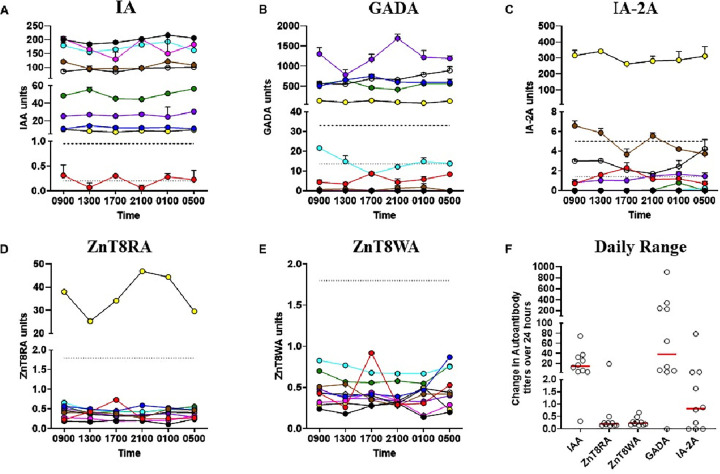
Diurnal Islet Autoantibody Variation Panels **A-E:** Antibody titers from 10 individuals with type 1 diabetes across time. Data from each individual are colored differently and connected across time. Harmonized (dashed lines; for IA, GADA and IA2A only) and local thresholds (dotted lines; based on assay performance at the Alistair Williams Antibody Facility, University of Bristol) are shown. **Panel F:** Daily intra-participant ranges are plotted for each antibody. Median antibody range is indicated by horizontal lines.

Having observed antibody isotype-specific immunoglobulin variations, we then asked whether islet Aabs also varied with time of day. We found appreciable fluctuations over the course of the day within some T1D individuals (Figs. 2A-E). A summary measure of diurnal variation for each participant (within-participant autoantibody range (maximum-minimum titer) during the 24-hour period) is shown in Fig. 2F. This daily difference was variable between individuals with some having zero change across time while other individuals had very large changes. For example, the daily difference in GADA was zero for one person while as great as 900 units for another. Similar observations were made for IA, ZnT8RA and IA-2A. “Typical” (i.e., median) daily differences in Aab titer were approximately 19 units for IA, 30 for GADA and 0.6 for IA-2A. Daily ranges of the zinc transporter Aab were typically small, yet one individual had an exceptionally large range for ZnT8RA (approximately 20 units). This individual was the only one who was ZnT8RA positive in the study cohort.

For several individuals, IA-2A, GADA and IA “detection” (i.e., titer exceeds threshold representing upper percentiles in a population not having T1D) changed across the day. To investigate whether these changes could be attributable solely to inter-assay variation we compared the diurnal CV of each individual to the local laboratory QC inter-assay CV (Appendix Fig. 3). In each case, the diurnal CV exceeded inter-assay CV several fold: IA-2A = 2.07 fold (subject 1), GADA = 2.14 fold (subject 6) and IA = 4.34 fold (subject 1). Hence, we conclude that in some individuals, diurnal assay variation exceeds inter-assay variation.

**Figure 3:**
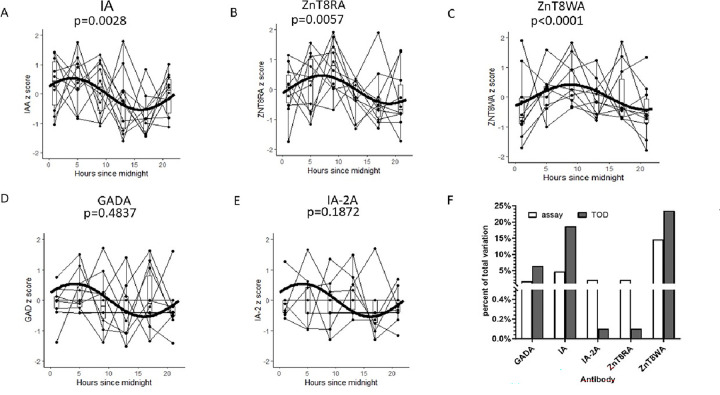
Circadian Aab Variation Panels **A-E:** Antibody titer data from 10 individuals with type 1 diabetes were standardized by “Z-scores” to reduce inter-individual variations and plotted across time and connected by fine lines. Bold superimposed sinusoidal single-phase curves were fit using the COSINOR method and depict circadian patterns with p-values. **Panel F:** Percent total variation attributable to intra-assay variation and to Time of Day (TOD) variation.

We also determined that IA and both ZnT8RA and ZnT8WA had statistically significant circadian variation, while GADA and IA-2A did not reach significance (Figs. 3A-E). Estimates of the amplitude and “Acrophase” (time of peak) circadian parameters of these autoantibodies are reported in Appendix Table 2.

To understand the contribution time of day has on islet Aab measurements, we performed a “Variance Components” analysis of sources of variation in the raw data (not Z-scores)-Figure 3F. Here, we found that the greatest source of variation in autoantibody titer was always differences between individuals; however, the variation attributable to the time of day the sample was taken (“TOD” in the figure) accounted for a greater proportion of assay variation than did intra-assay variation for GADA, IA and ZnT8WA.

Appendix Table 3 reports the results from a comparison of Aab precision (Standard Deviation) under time-restricted sampling to sampling that occurred randomly across the “clinical day” (9:00 to 17:00). Even with the small size of this study, there is evidence that restricted-time sampling can significantly increase Aab precision: Sampling at 13:00 led to reductions in Aab SD of 23%, 9% and 34% in GADA, IA and ZnT8RA, respectively.

In sum, we conclude that Aab titer and detection rates vary significantly across the day in some individuals to the extent that it should be controlled in order to increase Aab measurement precision and detection by future studies. In the next section we investigate the practical significance of diurnal Aab variation in a large clinical population and introduce a method to control diurnal Aab variation through restricted time sampling.

### Sampling Time Study

Having found significant diurnal variation in some islet autoantibodies, we then analyzed Aab collected from 705 individuals who each had a single blood draw at varying times of day (raw data are plotted in Appendix Fig. 2). Demographic characteristics of these individuals are presented in Table 1. Briefly, this study population was balanced in composition of sex, mostly White (Caucasian), had type 1 diabetes for a median of 3 years (0–59 years) and a median age of 20 years (range: 3–76 years).

**Figure 4:**
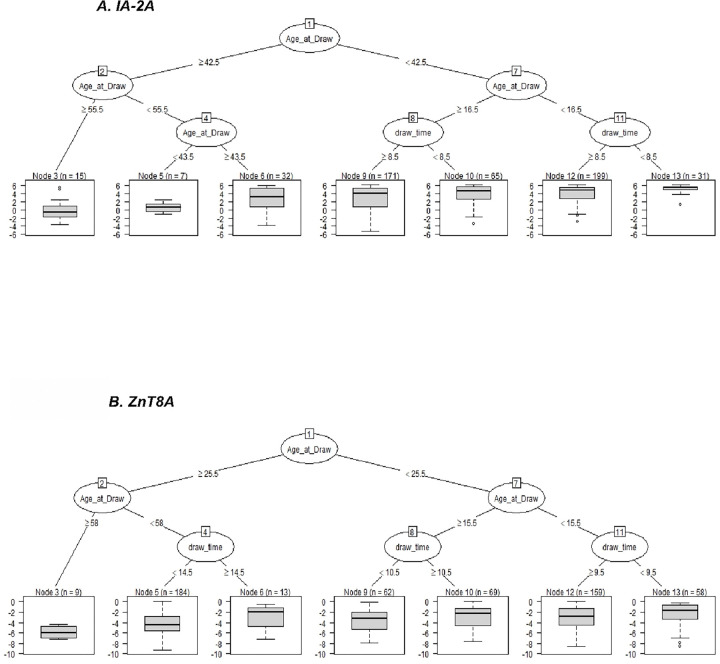
Age and Time-of-Day (TOD) Subgroups Identified by Machine Learning Results are presented for IA-2A (upper) and ZnT8 (lower). Diagrams for IA and GADA appear in Appendix Material. Top part of diagram of each figure presents the decision tree created by machine learning (CART). For example, IA-2A subjects younger than 42.5 years are split into two groups: those older than 16.5 years and those younger than 16.5 years. Subjects are then split in each of these branches based on TOD of sampling: After 08:00 vs before 08:00 (as we are using hours as our time unit, “<8.5” translates to “≤08:00, etc.). Lower part of diagram present boxplots of Ln IA-2A titers. Median titer is represented by horizontal bar. In each case when mean Ln IA-2A TOD differences were statistically significant (p<0.05 by ANOVA), mean titer was higher when sampled earlier.

We took a machine learning strategy to identify whether time of day would be a significant factor in subgrouping participants by mean log Aab titers. Here we found that age was the biggest factor in contributing to Aab levels; however, second to this was time of day (“TOD”). This indicated that TOD does have significant impact on Aab mean log titer for IA-2A and ZnT8A (Fig. 4) but not for GADA. IA did have significant combinations but is not discussed further owing to the possible confounding of the time of insulin supplementation with time of sampling: See Appendix Fig. 1 for IA and GADA.

We then statistically compared mean log titers between TOD subgroupings within age groups using ANOVA. Statistically significantly different results are presented in Table 2. In most subgroupings, sampling earlier in the day led to significantly greater mean log titers than sampling later in the day. For IA, sampling at or before 14:00 resulted in a 2.64-fold increase in titer than when sampling afterward (p = 0.0097) in children 14–19 years old. Sampling at or before 08:00 led to increased IA-2A titers both in those 16 years or younger (fold change = 3.49, p = 0.0051) and those 17–42 (fold change = 2.03, p = 0.0369). In individuals 15 years or younger, ZnT8A sampling at or before 09:00 also led to significantly increased titer detection (fold change = 2.01, p = 0.0243).

Exceptions to the rule of sampling earlier also occurred. In individuals 44 years or older, sampling at or before 09:00 resulted in a 0.44-fold change (p = 0.0459) in IA titer. Conversely, it can be concluded that sampling after 09:00 resulted in a 2.27-fold increase. Similarly, in persons 26–57 years old, sampling at or before 14:00 resulted in a 0.31-fold change (p = 0.0433) in ZnT8A titer. Conversely, this implies a 3.23-fold increase when sampling after 14:00.

We then compared rates of autoantibody detection in the subgroups described above (Table 3). IA-2A detection rates were significantly increased when sampling at or before 08:00 for participants 16 or younger (91% vs 64%, p = 0.0022) and participants 17–42 (61% vs 48%, p = 0.0442). An increase in ZnT8A detection was observed in participants 15 or younger when sampling at or before 09:00 (71% vs 54%, p = 0.0134); however, for participants 26–57 years of age, ZnT8A detection was greater in magnitude but not found statistically significant when sampling later than 14:00 than earlier (44% vs 28%, p = 0.1332). Age and TOD sampling did not significantly alter IA detection rates despite the statistically significant differences found in IA titer.

To summarize, our sampling time study established that both Aab titer and detection vary by time of day within specific age groups and this variation can be clinically relevant. Restricting the time of sampling appears to increase yield for specific age groups.

## Discussion

We found that immunoglobulins and islet autoantibodies exhibit wide variation across the day in some individuals with stage 4 type 1 diabetes and which is sometimes large enough to alter detection. From the population perspective, we conclude that immunoglobulin isotypes and IA and ZnT8A exhibit significant circadian patterns in stage 4 type 1 diabetes. In a large and independent population, machine learning identified sampling times within certain age groups which increase both mean titers and detection rates for IA-2A and ZnT8A. Therefore, we conclude that restricting the time period of sampling peripheral blood would reduce a source of Aab variation that arises from diurnal changes, thereby increasing the precision of Aab measurement which subsequently would increase the power of clinical studies testing new interventions in established T1D. In addition, we also conclude that time-restriction based on the age of the individual will increase detection of some Aab’s and therefore increase study yield as well. Time-restriction therefore can potentially improve the use of Aabs as biomarkers for the development and monitoring of disease-modifying therapies and in developing criteria for selection of persons most likely to benefit from islet transplantation. This will require further studies to fully address.

It should be noted that the machine learning algorithm sought to determine sampling times which created the greatest difference in mean log titer and that it was agnostic to detection rates. Therefore, we conclude that our findings related to increased detection rates are not inflated due to bias in model overfitting. We have, however, evaluated the statistical error in our model-based estimates of mean log titer using 10-fold Cross-Validation and these data are reported in the Appendix which contains a print-out of the CART output for each Aab.

Regarding our findings of optimal sampling times, one possible explanation is confounding of our age-TOD subgroupings with diabetes duration. However, the groups did not significantly differ in mean duration (Appendix Table 4).

Diurnal variation of the human immune system is well established[20]. This variation is thought to be driven largely by tissue-resident circadian clock genes and the entrainment of the organism to external environmental cues via the suprachiasmatic nucleus. Islet cell circadian clocks regulate several cellular β-cell processes, including insulin exocytosis[21, 22]. Therefore, it is possible that circadian variation of autoantibodies reflects circadian variation in antigen release that is created by time-varying, circadian-governed islet cell insulin exocytosis. Presentation of antigen to B-cell leads to their expansion and so, we would expect that B-cell populations should rise and fall during the day and that is exactly what we have observed in our prior research within this population[19].

Of course, the diurnal variation of IA might also in part reflect the influence of exogenous insulin administration. Fuchtenbusch[23] found that exposure to exogenous insulin promoted IgG1 and IgG4 responses to insulin but not to other islet autoantigens (e.g., GAD and IA-2). Yet, since our data did not include timing of exogenous insulin administration for each individual, we are unable to investigate whether this was a confounding factor in our study and represents a limitation.

Since autoantibodies have a long half-life, the changes in Aab levels in the peripheral blood are interesting. This could be related to Fc receptor binding, as Fc receptors are diurnally expressed[16] and can remove antibodies from circulation, preventing their degradation, and extending their survival[24, 25]. Furthermore, islet Aabs have been shown to bind Fc receptors[26].

An alternative explanation for diurnal variation is variation in hydration; however, a study assessing circadian changes in the blood, saliva and urine of healthy individuals asked to intake high or low water volumes, found no circadian impact of water intake on plasma osmolarity, while urinary markers were significantly affected in a circadian manner[27]. Furthermore, individuals fasting for 12 hours from food and drink also showed plasma and serum volume remained unchanged; however, once again urinary markers were altered[28]. Together, these studies suggest changes in hydration would not fully explain the diurnal differences we have observed in this study.

The role of assay variation needs to be considered when interpreting our findings. We observed that more than half of subjects had diurnal variation exceeding lab-based expectations in GADA, IA-2A and ZnT8WA. Hence, we conclude that diurnal variation can often be greater than that explained by simple inter-assay variation.

Regarding changing Aab detection across the day, it is possible that this variation is due to statistical variation of the assay itself (intra-assay variation). Intra-assay CVs (SD) near threshold values were: GADA = 22.94 (4.98); IA-2A = 29.50(1.70); IA = 22.23(0.14); ZnT8RA = 21.02(1.44) and ZnT8WA = 20.38(0.55). Therefore, since in all cases of altering Aab detection titers were close to threshold, the changes in Aab detection might be due solely to statistical variation. However, the Variance Components Analysis suggests that TOD-attributable variation accounted for a greater proportion of the observed diurnal variation than did intra-assay variation for GADA, IA and ZnT8WA In sum, our data documents that Aab detection can vary across the day and, even if due solely to statistical variation, this variation needs to be considered for subjects with assay values near threshold. We recommend that subjects having Aab within 2 standard deviations of threshold should be retested.

Limitations of our two studies need to be considered when interpreting our findings. Our “Diurnal Variation Study” was based on a small sample of individuals living with Stage 4 type 1 diabetes; however, this is a common sample size found in human circadian studies which is a practical restriction driven by the expense and burden that is imposed on participants who need to spend 24 hours in a research unit. On the other hand, the sample size was large enough to detect significant circadian patterns in several autoantibodies. We suspect that a larger sample size might have successfully found circadian patterns in GADA and IA-2A. In contrast, our “Sampling Time Study” had a much larger sample size but was cross-sectional so that each participant contributed only one observation. Thus, we were not able to evaluate recommendations for TOD sampling on individual subjects across time. We decided not to include diabetes duration in our CART model as we wanted to limit the complexity of our tree to 3 levels in order to facilitate interpretation. We did however compare duration between the groups identified by CART (Appendix Table 3) and found no significant difference in mean duration between the age-TOD subgroups. We therefore conclude that the absence of duration in our CART model did not lead to confounding in the relationship between log titer and the age-TOD subgroups. Yet, we acknowledge that the absence of duration in our models is a limitation. In addition, although there was no significant correlation between observed sampling times and diabetes duration, we did find that age was significantly and negatively correlated with sampling time (Spearman Rank Correlation=−0.122, p = 0.0011). Thus, we found that younger persons tended to have later sampling times. In addition, age was found to be negatively correlated with IA, IA-2A and ZnT8 log titers (all p < 0.001) suggesting that log Aab titers are higher in younger individuals than older. Since younger individuals had later sampling times with higher log Aab titers we would expect that this possible confounding would bias our findings toward determining that later sampling times were beneficial, which was not the case in most TOD-age combinations. Our study is limited to Aab measurement by Radiobinding Assay and our findings and conclusions do not necessarily transfer to other platforms. Finally, it remains to be determined the extent to which diurnal variation is due to intrinsic assay variations vs. true (biological) diurnal variation. Yet, even if it is the case that the sole cause of the observed diurnal variations is simply assay variation, we have nonetheless established empirically that individuals can gain/lose antibodies across a 24-hour period. This discovery should motivate the adoption of multiple measurement of titer when values are near threshold. However, given previous literature on diurnal variation of the human immune system and the fact that our small clinical study was able to discern circadian patterns in IA, ZnT8RA and ZnT8WA we conclude that diurnal variation is sometimes not simply due to assay variation. We look forward to future efforts addressing this issue.

We conclude that autoantibodies should be measured during a restricted-time window in order to reduce the impact of diurnal variation and thereby increase study sensitivity and yield. We also suggest that a commonly-adopted “standardized sampling time window” would ensure comparability of Ab findings across studies. An alternative to time-restriction approach would be to have a threshold for autoantibody positivity which changes with the time of day to reflect population-level diurnal changes in autoantibody distribution.

Our conclusions need to be tested independently. We look forward to further refinements of this innovative approach to autoantibody measurement and to developing and testing standardized time sampling in type 1 diabetes screening populations, in whom we anticipate that optimal sampling times may be different from a stage 4 type 1 diabetes population.

## Methods

Herein we report findings from two studies. First, a prospective study of the range of islet autoantibody and immunoglobulin daily variation within individuals and to determine whether Aab titers followed a circadian pattern, a particular form of diurnal variation in which there is a single peak and trough of Aab titer over a 24-hour period and the variation over the day can be represented by a cosine curve (“Diurnal Variation Study”). Second, having found significant diurnal variation, we then sought to understand its practical implications in an independent retrospective, cross-sectional study of the effect of time of sample collection on Aab titer and detection (“Sampling Time Study”).

Diurnal Variation Study

### Study participants

Participants were between 18–40 years of age, had type 1 diabetes treated with insulin for at least 12 months without any other clinically significant diseases or illnesses, weighed at least 110 pounds (49.9 kg), and were willing to abstain from alcohol, maintain a regular sleep schedule for 1 week prior to the study visit and spend 24-hours in the research facility. Exclusion criteria included significant acute/chronic illnesses, shift work, prior or concurrent immunomodulatory drug exposure and recent travel between time zones. After providing written informed consent, participants underwent a medical history check and physical examination. Beginning at 09:00, peripheral blood was collected by venipuncture every 4-hours over a 24-hour period. Participants had free access to food and water, with lights turned off at 23:00. Diet was not controlled to reflect a real-world setting. All participants provided consent. The Indiana University Institutional Review Board approved all study procedures, and all research was performed according to their guidelines and in accordance with the Declaration of Helsinki. We also analyzed immunoglobulins in persons who did not have type 1 diabetes (“historical controls”). Details about these individuals were previously reported[19].

### Immunoglobulin assay measurements

Total IgM, IgA, IgA1, IgA2, IgG, IgG1, IgG2, IgG3 and IgG4 antibodies were investigated from plasma samples from the Diurnal Variation Study participants. Samples were diluted 1:100–1:2000 in sterile PBS. 96-well high-binding ELISA plates (Greiner) were coated with an anti-human isotype-specific antibody in 0.1M NaHCO_3_ buffer. All plates were coated overnight at 4°C. Wells were washed in PBS/0.05% Tween-20 four times prior to blocking in PSB/1% BSA for 1h. Samples were added to the plate in duplicate and incubated at room temperature for 2h. Wells were washed four times, anti-isotype-specific alkaline phosphatase-conjugated detection antibodies were added (1:1000) and samples were incubated for a further 2h. Wells were washed again prior to addition of substrate (p-Nitrophenyl phosphate), which induced an enzymatic reaction with a detectable color change. The enzymatic reaction was stopped using 1M NaOH and the optical density was measured on an ELISA plate reader at 405nm. Concentrations were determined from the standard curve. All antibodies and standards were obtained from Southern Biotech, except for the standards for human IgA1, IgA2, IgG2 and IgG4, which were purchased from Merck.

### Islet Autoantibody radiobinding assay measurements

Antibodies against insulin (IA), and autoantibodies to glutamic acid decarboxylase 65 (GADA), tyrosine phosphatase-related islet antigen 2 (IA-2A) and zinc transporter 8 antibody (ZnT8) (both arginine (ZnT8RA) and tryptophan (ZnT8WA) variants) were measured in duplicate EDTA plasma samples by radiobinding assays (RBA). Antibodies to insulin are referred to as antibodies rather than autoantibodies because participants received exogenous insulin and thus it is unclear whether these are true autoantibodies. Further information about the RBA assays can be found in the Appendix. The data analyzed below are the within-participant average of the titers from two samples. All antibody assays were successfully completed without missing data.

### Statistical analysis

Diurnal antibody changes for each individual were summarized with daily ranges (daily maximum-daily minimum). Variance Components analysis was used to estimate the percent variation in each antibody attributable to between-participant variation, time-of-day variation and assay variation. Statistical variation of antibodies over time was characterized using the nonlinear “Cosinor” model[29] after transforming the data to participant-specific z-scores. This model (with random effect for individual) fitted a single-phase, 24 hour-period sinusoidal curve to the data and provided statistical significance of the fitted model as well as statistical estimates and 95% confidence intervals of the time of highest Ab titer (Acrophase) level. All analyses were carried out in GraphPad Prism 10.0 (La Jolla, CA), SAS V.9 (Cary, NC) or R (R Core Team, 2021). A p-value < 0.05 was considered statistically significant.

Potential for increased titer accuracy via time-restriction was investigated with Bootstrapped simulation of random time selections for each individual versus sampling at one of three specific times (9:00, 13:00, 17:00). The ratio of assay standard deviation (SD) (restricted-time SD/random-time SD) was estimated in 10,000 Bootstrapped samples and median SD ratio with bootstrapped 95% confidence intervals summarized.

Sampling Time Study

We also conducted a retrospective cross-sectional analysis of persons with established type 1 diabetes enrolled in the Benaroya Research Institute (BRI) Diabetes Registry and Repository. The study enrolls participants over the age of 1 year who have been diagnosed with type 1 diabetes; some participants agreed to undergo mixed meal tolerance testing as part of the study. The study was approved by the BRI Institutional Review Board (IRB #10024) all research was performed according to their guidelines and in accordance with the Declaration of Helsinki. Participants or guardians provided written informed consent, with additional assent for those aged 7–11 years. Sampling time was available (median sampling time = 11:00, range = 7:00–16:00) from each individual who contributed one sample. Radiobinding islet autoantibody measurements were conducted at the Barbara Davis Center.

### Statistical Analysis

We transformed the autoantibody titer measurements to logs to better meet the statistical requirements for analysis by ANOVA. In addition, we rounded sampling times (recorded in hours and minutes) to hours for analysis in order to reduce possible variance inflation due to measurement error. We then used machine learning to identify sampling times (optimal sampling time or “TOD”) that created the greatest mean difference between subgroups identified by age and sampling time combinations. Our machine learning approach used the “Classification and Regression Tree (CART)” algorithm as implemented in the RPART and PARTYKIT packages in R (R Core Team (2024)).

We then statistically compared the mean log titers between Age-TOD subgroups using ANOVA. In addition, we compared autoantibody detection rates (% detected) between the subgroups using the chi-square test. In all analyses, p-values < 0.05 were considered statistically significant.

## Supplementary Material

Supplementary Files

This is a list of supplementary files associated with this preprint. Click to download.

• SupplementalMaterial.docx

## Figures and Tables

**Table 1:** Demographic Characteristics-Sampling Time Study

Variable	N	Percent
**Sex: Female**	350	52.2%
**Race: White, Caucasian**	533	93.2%
**GADA+**	495	70.2%
**IA+**	636	90.2%
**IA-2A+**	383	54.3%
**ZnT8+**	306	43.4%
Variable	N	Median (IQR†)
**Age (yrs.)**	704	20.00 (21.00)
**T1D Duration (yrs.)**	705	3.10 (8.70)
**GADA titer**	705	75.62 (373.58)
**IA titer**	705	0.22 (0.62)
**IA-2A titer**	705	14.65 (210.45)
**ZnT8 titer**	705	0.01 (0.12)

† Interquartile Range.

**Table 2:** Optimal Sampling Times

Aab (Ln)	Age (yrs.)	TOD† Mean Ln Aab		Earlier-Later Mean Difference	Earlier/Later Fold Change	p-value
**IA**						
		***<=14:00***	***> 14:00***			
	**14–19**	−1.39	−2.35	0.97	2.64	0.0097
		**<=9:00**	**> 9:00**			
	**>=44**	−2.48	−1.65	−0.82	0.44	0.0459
**IA-2A**		**<=8:00**	**> 8:00**			
	**<= 16**	5.28	4.03	1.25	3.49	0.0051
	**17–42**	3.86	3.15	0.71	2.03	0.0369
**ZnT8A**		**<=9:00**	**> 9:00**			
	**<=15**	−2.38	−3.05	0.70	2.01	0.0243
		**<=14:00**	**> 14:00**			
	**26–57**	−4.24	−3.07	−1.17	0.31	0.0433

† TOD=optimal time of day for sampling.

**Table 3:** Detection Rates by Time of Day (TOD)

Autoantibody	Age yrs.	TOD/Percent Detection by TOD†		p-value
**IA**	**14–19**	***≤ 14:00***	***> 14:00***	
		94.8% (89.5%, 96.9%)	87.5% (67.6%, 97.3%)	0.1776
	**≥ 44**	**≤ 9:00**	**> 9:00**	
		82.6% (61.2%, 95.1%)	92.2% (82.7%, 97.4%)	0.1957
**IA-2A**	**≤ 16**	**≤ 8:00**	**> 8:00**	
		90.6% (75.0%, 98.0%)	63.5% (57.2%,69.4%)	0.0022
	**17–42**	**≤ 8:00**	**> 8:00**	
		60.9% (49.9%,71.2%)	48.3% (41.7%,54.9%)	0.0442
**ZnT8A**	**≤ 15**	**≤ 9:00**	**> 9:00**	
		71.4% (58.7%,82.1%)	53.7% (46.3%,60.9%)	0.0134
	**26–57**	**≤ 14:00**	**> 14:00**	
		27.8% (22.3%,33.8%)	44.4% (21.5%,69.2%)	0.1332

† 95% confidence interval on detection appears in parentheses.

## Data Availability

Data will be shared upon reasonable request to the corresponding authors.
